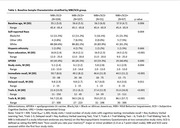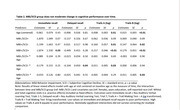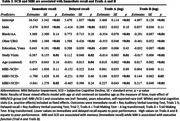# Mild Behavior Impairment with Subjective Cognitive Decline is associated with impaired cognitive function

**DOI:** 10.1002/alz70857_104698

**Published:** 2025-12-25

**Authors:** Carol A. Van Hulle, Mary F. Wyman, Carey E. Gleason, Sterling C Johnson, Sanjay Asthana, Cynthia M. Carlsson, Barbara L. Fischer

**Affiliations:** ^1^ Wisconsin Alzheimer's Disease Research Center, University of Wisconsin‐Madison School of Medicine & Public Health, Madison, WI, USA; ^2^ Division of Geriatrics and Gerontology, Department of Medicine, University of Wisconsin School of Medicine and Public Health, Madison, WI, USA; ^3^ Geriatric Research, Education and Clinical Center (GRECC), William S. Middleton Memorial Veterans Hospital, Madison, WI, USA; ^4^ Division of Geriatrics and Gerontology, Department of Medicine, University of Wisconsin‐Madison, School of Medicine & Public Health, Madison, WI, USA; ^5^ Wisconsin Alzheimer's Institute, University of Wisconsin School of Medicine and Public Health, Madison, WI, USA; ^6^ University of Wisconsin School of Medicine and Public Health, Madison, WI, USA; ^7^ Wisconsin Alzheimer's Disease Research Center, School of Medicine and Public Health, University of Wisconsin‐Madison, Madison, WI, USA; ^8^ Department of Medicine, University of Wisconsin‐Madison School of Medicine and Public Health, Madison, WI, USA; ^9^ Wisconsin Alzheimer's Disease Research Center, University of Wisconsin School of Medicine and Public Health, Madison, WI, USA; ^10^ Department of Neurology, University of Wisconsin‐Madison, School of Medicine and Public Health, Madison, WI, USA; ^11^ VA Geriatric Research, Education and Clinical Center (GRECC), William S. Middleton Memorial Veterans Hospital, Madison, WI, USA

## Abstract

**Background:**

Mild Behavior Impairment (MBI), a syndrome referring to neuropsychiatric symptoms emerging in mid to late life, is common prior to dementia onset and may have a impact on cognitive decline. Subjective cognitive decline (SCD), the subjective perception of cognitive decline in the absence of objective cognitive impairment, is a potential risk factor for conversion to dementia. Evidence suggests combined SCD and MBI represents greater risk for conversion to cognitive impairment than either alone. For this study we explored the impact of SCD and MBI on cognitive performance in a relatively young, cognitively unimpaired sample.

**Method:**

Participants from the Wisconsin Alzheimer's Disease Research Center were included if cognitively unimpaired at baseline. MBI was defined by a study partner endorsing any item on the Neuropsychiatric Inventory Questionnaire at two consecutive study visits. SCD was identified by reporting minor or major memory problems on the question, “How would you rate your memory?” Participants were categorized based on the presence or absence of MBI and SCD in the first four study visits. Cognitive outcomes included the Rey Auditory Verbal Learning Test Immediate and Delayed Recall and Trail Making Tests A and B.

Linear mixed effects models were used to test the association between MBI/SCD group and cognitive performance over time. We examined the main effects of MBI/SCD group on overall cognitive performance after removing non‐significant interactions.

**Results:**

*N* = 309 participants (mean age 56.3, range 45‐66) provided *N* = 1,365 observations (see Table 1 for sample characteristics). Participants with SCD were more likely to meet criteria for MBI (χ^2^[1] = 8.7, *p* = .001). The interaction between SCD/MBI group and time was inconsistent across outcomes and did not survive multiple correction (Table 2). Participants in the MBI+/SCD+ group had poorer overall performance on immediate recall while participants experiencing MBI with and without SCD had poorer overall performance on Trails A and B (Table 3).

**Conclusion:**

These results suggest MBI alone may function as a marker for impaired executive function in middle aged participants at the earliest stages of disease. When seen in combination with SCD, MBI appears to portend impaired memory. Future work will investigate co‐occurring pathology.